# The impact of financial development, health expenditure, CO2 emissions, institutional quality, and energy Mix on life expectancy in Eastern Europe: CS-ARDL and quantile regression Approaches

**DOI:** 10.1016/j.heliyon.2023.e21084

**Published:** 2023-10-31

**Authors:** Elvira Nica, Adela Poliakova, Gheorghe H. Popescu, Katarina Valaskova, Stefan Gabriel Burcea, Andreea-Ligia Drugau Constantin

**Affiliations:** a Department of Administration and Public Management, Faculty of Administration and Public Management, Bucharest University of Economic Studies, Piața Romană, 6, Romania; b Department of Economics, Faculty of Operation and Economics of Transport and Communications, University of Zilina, Zilina, Slovak Republic; c Department of Finance, Banking and Accounting, Faculty of Finance, Banking and Accounting "Dimitrie Cantemir" Christian University, Bucharest, Romania

**Keywords:** Eastern europe, Institutional quality, Financial development, Life expectancy, Health outcomes

## Abstract

In recent years, the interrelationships between the environment, energy, and health have received a growing amount of attention due to their substantial impact on the health of humans. By examining what influences Eastern Europeans' longevity between 1990 and 2021, this study hopes to contribute to this field of study. Energy consumption, health expenditure, pollution, institutional quality index (IQI), financial development, and other attributes profoundly impact human health. Because of the extensive network of commerce, tourism, education, religion, and treaties connecting East European countries, tests for cross-sectional dependence (CSD) and slope heterogeneity (SH) are utilized. After verifying the CSD and SH issues, the study uses the second generation's unit root and cointegration tests. As the previous test indicates, a new panel method, the cross-sectional autoregressive distributive lag (CS-ARDL) model, is required, as conventional estimations are inappropriate. The Quantile Regression (QR) method is also applied to check robustness. This study indicated that increased health expenditure, renewable energy consumption, and IQI improves health outcomes in Eastern European nations. There was a good connection between renewable energy consumption and health benefits, the study concluded. Though financial development positively impacts life expectancy, the impact is insignificant. On the other hand, the study also shows that CO_2_ emissions and fossil fuel consumption decrease life expectancy. These results are consistent with those obtained using the QR method. To enhance health outcomes, it is necessary to take measures to raise health spending, increase the use of renewable energy, and foster financial development over the long term. On the other hand, Eastern European nations need to shift their attention from fossil fuels to renewable energy.

## 1 Introduction

The relationship between the environment, energy, development, and health is crucial to sustainable development and substantially impacts human well-being. Eastern Europe, an area with distinct political and historical traditions, has seen substantial economic, environmental, and social transformations in recent decades [1]. By delving into the relationships between the environment, energy, economy, and health, this study hopes to understand better the factors contributing to Eastern Europe's relatively low life expectancy. This study focused on a few Eastern European nations to examine the correlation between rising living standards, increased energy consumption, and improved health. The goals of this study are to (i) ascertain the impact of financial development on health outcomes, (ii) examine the connection between the use of fossil fuels and renewable energy and health outcomes, and (ii) evaluate whether or not there is a statistically significant relationship between CO2 emission and health consequences.

The target to "secure good health and encourage well-being for all people of all age groups" is one of the SDGs, which seem to be priorities that every country agreed to strive to achieve by 2030 [2]**.** Precisely, it aims to "significantly reduce worldwide infant mortality rates" in addition to "drastically boost health financing" [3]**.** One of the main factors in achieving such essential points of the SDGs is public spending on health [4]. Healthcare spending is critical to health systems' ability to maintain and enhance human well-being; without funding, qualified and suitable medical workers would be unable to offer a job, medical technology would not be accessible, and public health or disease prevention would not occur [5]**.** Health spending demonstrates the population's overall consumption of health-related services and goods globally [6]**.** Financing in the health system leads to healthier lifestyles, job creation, improved political and social reliability, and economic expansion and development [6]**.** Health spending records for 7 % of GDP in low-income countries and 6 % in middle-income countries [7]**.** The healthcare sector has expanded far faster than the economy during the past 15 years [8].

Life expectancy has recently been in the news due to its stagnation or decline in Central and Eastern Europe [9,10]. Death rates from cardiovascular disease are on the rise, which is primarily responsible for the current poor mortality trends in Central and Eastern Europe [11]. Low-quality food, excessive alcohol use, a lack of access to sufficient health care, unemployment, poverty, and psychosocial stress are all variables that contribute to these diseases, as do the political and economic situations during the communist and post-communist eras. It doesn't have to be this way. Since the decline in mortality rates during working age has slowed while mortality rates for older people have continued to fall, many documented cases of increased lifespan variation are happening alongside increases in life expectancy [12]. No systematic analyses have examined the variance in lifespan during declining life expectancy. Because mortality decreases over young ages have outpaced mortality declines at later generations, most deaths have been compressed into a smaller age window, making the ages at death more predictable (i.e., lifespan variation has decreased) along with increases in life expectancy [13]. Energy consumption is correlated with increased economic activity and, consequently, increased CO_2_ pollution. Many earlier studies have yet to address the connection between energy consumption, pollution, IQI, economic growth, and health outcomes, even though the impact of energy growth on health outcomes in this region has been identified [14]. Bloom et al. [15] examined the link between people's health and a nation's GDP. Their findings show that increasing GDP positively impacts life expectancy, and the results are significant. Consistent evidence indicates a positive relationship between a healthy population and economic growth.

The research is motivated by the need to understand the complex interplay of environment, economics, development, energy, and health outcome in Eastern Europe. This region has a long history of environmental degradation, which has significantly impacted human health. Similarly, changes in Eastern Europe's political, economic, and social conditions over the past few decades may have affected health outcomes there. These nations' financial health, population, healthcare costs, IQI, and energy consumption have increased [16]. The percentage of GDP spent on healthcare in Western European countries increased from 7.7 % in 2000 to 10.5 % in 2009 [17]. The governments of Eastern Europe are rapidly developing into global economic superpowers. Economies in the East are growing by over 6 % annually on average. Therefore, it is crucial to ascertain the effect on health outcomes. In recent years, there has been a growing focus on the need to improve environmental quality in Eastern Europe, and this research will help to inform these efforts. The study is important in Eastern European countries because some of the region's countries face many environmental, energy, and health challenges. Albania, Bosnia, Cyprus, and Kosovo have faced health challenges [[18], [19]]. The paper contributes to the current literature due to these presuppositions: The first part of the study examines how CO2 emission, financial development, healthcare spending, institutional quality, and energy utilization have affected life expectancy in Eastern Europe from 1996 to 2021. Furthermore, extensive contact exists between Eastern European governments due to trade, religion, bilateral collaboration, and cultural exchange. According to the data, CSD and SH are both serious issues. Cointegration tests, cross-sectional autoregressive distributive lag (CS-ARDL) tests, and second-generation cross-sectional unit root tests were used to analyze the data and find solutions. In researching factors affecting people's lifespans, the relatively recent econometric approach known as the conditional stationary autoregressive distributed lags (CS-ARDL) method is invaluable. This technique estimates the short-run links between the selected variables and life expectancy and identifies the long-term linkages between these two variables. Smith's [20] health production model serves as the theoretical foundation for this investigation into the effects on health outcomes. The paper concludes with recommendations on how policymakers might consider economic growth, population size, energy use, and carbon dioxide emissions. Increases in health spending and decreases in pollution, for example, are only two examples of how this information might be used to improve people's health.

The research will help address these challenges by better understanding the complex relationships between environment, energy, and health. This understanding can then be used to develop policies and programs that can help to improve public health in Eastern Europe.

This research identifies the following significant hypotheses.

I. Financial Development Hypothesis or H1: Higher levels of financial development positively impact life expectancy in Eastern Europe, as improved access to financial resources can enhance healthcare infrastructure and services.
II. Health Expenditure Hypothesis or H2: Increased health expenditure is positively associated with higher life expectancy in Eastern Europe, indicating that investments in healthcare resources, facilities, and services contribute to improved population health outcomes.
III. CO2 Emission Hypothesis or H3: Higher levels of CO2 emissions negatively affect life expectancy in Eastern Europe, as environmental pollution and exposure to harmful pollutants can have adverse health effects, leading to decreased life expectancy.
IV. Institutional Quality Hypothesis or H4: Better institutional quality positively influences life expectancy in Eastern Europe, suggesting that strong governance, efficient public administration, and effective policies contribute to improved health outcomes and overall well-being.
V. Renewable Energy Hypothesis or H5: Greater utilization of renewable energy sources positively affects life expectancy in Eastern Europe, reducing environmental pollution and potential health hazards associated with fossil fuel combustion.
VI. Fossil Fuels Hypothesis or H6: Higher reliance on fossil fuels negatively impacts life expectancy in Eastern Europe, as it is associated with increased air pollution, respiratory diseases, and other health risks.

These hypotheses form the basis for investigating the complex interconnections between environmental factors, energy sources, health indicators, and life expectancy in Eastern Europe.

Section 2 is a literature review that follows the introduction. Following that is section 3, which contains the data, the theoretical framework, and the econometric models. In the fourth section, the findings and results are presented. The debate is presented in Section 5. The conclusion is shown in the following section 6. The section on policy recommendations follows just after the decision. Limitations and future research will be discussed afterward. This paper contains two supplementary materials. The glossary of abbreviations can be found in Appendix A. The countries of Eastern Europe are listed in Appendix B.

## 2 Literature reviews

The relationships between health, economic development, and energy consumption have been the subject of numerous studies. These analyses are performed on a global or regional scale.

Mankiw et al. [21] looked at the correlation between health and economic development as one component of human capital. Therefore, health capital is a typical production indicator, as stated by Mankiw et al. [21]. Jorgenson et al. [22] analyze the transition from communist command economies to market demand economies in Central and Eastern European (CEE) countries, focusing on the interplay between energy use, human well-being, economic growth, and human health. According to the findings, the quality of life in these countries improved dramatically as their energy intensity decreased and their energy efficiency rose. The results indicate future harmony among progress, health outcome, and the planet's health. Lucas [23] discovered a similar finding. There has been a lot of study into the correlation between energy use and economic growth. The article by Alharthi et al. [24] investigates the impact of environmental pollution on human health and the financial status of households in MENA countries. It also explores the potential of renewable energy to eliminate environmental pollution. The study provides insights into the complex relationship between pollution, health, and economic well-being, emphasizing the need for sustainable energy sources to mitigate ecological challenges in the region. According to research by Tutak and Brodny [25], transitioning to renewable energy sources increases GDP, decreases carbon dioxide emissions, and lessens dependence on traditional fuels in nearly every EU member state.

The long-term causality between renewable energy and healthcare expenditure was found by Mujtabe & Sahazad [26] in their study of OECD countries. Using various panel techniques, including two-stage least squares, random effects, fixed effects, pooled OLS, and the Generalized Method of Moments (GMM), Majeed et al. [27] investigated the association between renewable energy usage and health outcomes in 155 economies. Using renewable energy sources benefits health, as shown through empirical studies. Using renewable energy sources has been linked to a lower death rate and a longer typical lifespan. Sustainable energy helps control chronic diseases, lengthens life expectancy, decreases mortality and tuberculosis rates, and positively affects public health. Hanif [28] utilized the GMM approach to investigate the connection between various energy consumption habits and the quality of people's health in Sub-Saharan Africa. The research found that using fossil fuels (gas, coal, oil) for cooking and burning solid fuels (wood pellets, peat, charcoal, wood, and agricultural wastes) for heating significantly increased total birth rates. Furthermore, the statistics show that fossil and solid fuel use reduces life expectancy in Sub-Saharan African countries. The results indicate that economic development and using renewable energy sources like wind, sun, and water (which can reduce people's exposure to harmful pollutants) are linked to lower mortality rates and better total birth management.

Smits & Monden [29] used time series data from Turkey to analyze the correlation between GDP growth and healthcare expenditure. Using the Granger causality and Johansen cointegration test, the researchers discovered a strong positive correlation between economic growth and healthcare expenditure. Chen et al. [30] 2021 examined the relationship between life expectancy and 20 economic and environmental progress indicators in developing and developed nations. Multiple regression models were used to ascertain the impact of each hand on life expectancy, and the Pearson Correlation Coefficient was used to analyze the relationships between the factors. Life expectancy was positively correlated with per capita wealth in both high- and low-income countries. Miladinov [31], using a Full Information Maximum Likelihood model, investigated the correlation between financial expansion and infant mortality in the five nations vying for European Union admission. The study's findings also showed a correlation between higher income and reduced neonatal mortality rates, contributing to extended life expectancy. Wang et al. [32] used time series data from Pakistan to investigate whether or not there was a connection between rising living standards and longer lifespans. The study used the ARDL bound testing technique, and their findings confirmed that economic growth is correlated with increased longevity.

Similarly, to establish the direction of the causal relationship between energy consumption and health effects, Youssef et al. [33] employed the SUR method. The study's findings demonstrated a positive correlation between wellness and energy use. Wang [34] applied an exposure-response analysis to the question of how much our energy use impacts public health and the ecosystem. These results demonstrate the dual nature of the effects of energy consumption on human health. The changing relationship between economic growth, energy consumption, and health in Sub-Saharan Africa was analyzed by Arawomo et al. [35]. Analysis shows no significant relationship between energy consumption and health outcomes or between energy consumption and economic development. The relationship between energy consumption and health outcomes was established to be unidirectional by Smith et al. [36] but bidirectional by Youssef et al. [33]. The study's authors, Smith et al. [36], discovered a positive and negative correlation between energy consumption and health results. Therefore, it is clear that the association between outcomes and economic development has been thoroughly investigated within the framework of growth theories illustrated in the previous review of the literature. There is no indication that the connection between the two variables is bidirectional or that the impact could have the opposite direction [22]. Therefore, studies have yet to examine how rising wealth influences health. Despite the limited research, rising wealth has a mixed effect on health.

These empirical findings contribute to our understanding of the complex relationships between energy consumption, financial development, CO2 emission, IQI, and health outcome, highlighting the importance of sustainable energy practices for positive health outcomes. These analyses are performed on a global or regional scale. Here is a summary of the empirical studies on the relationship between health outcomes, economic development, CO2 emission, and energy consumption. Empirical studies have shown that energy consumption, financial development, CO2 emission, IQI, and health outcome are interconnected. Economic growth and renewable energy use are associated with improved health outcomes. Fossil fuel use and CO2 emission are related to adverse health outcomes. The relationship between energy consumption and health outcomes is complex and may be bidirectional. Policies promoting economic development and renewable energy use can positively affect health. These studies suggest that financial development, energy consumption, pollution, IQI, and health are interconnected. Policies promoting financial development and renewable energy use can positively affect health, while policies promoting pollution and fossil fuel use can negatively affect health.

This study aims to fill the "literature gap" in the existing body of research by examining the association between IQI, financial development, energy consumption, pollution, healthcare spending, and life expectancy. Although research on the effects of these factors on longevity has been undertaken in other parts of the world, studies focusing on Eastern Europe are scarce. Life expectancy-related associations in Eastern Europe may have been affected by the region's dramatic political, economic, and social shifts over the past few decades. As a result, more study is required to comprehend these connections in Eastern Europe fully. CSD and SH were not included in the prior studies. Eastern European countries are intricately linked in education, tourism, religion, and trade, making the CSD exam crucial. All Eastern European countries are varied in size, GDP, energy consumption, and other statistics, making the SH test all the more crucial. A shortage of studies also uses the CS-ARDL method to investigate the connections between these factors and longevity. The CS-ARDL approach is novel and has yet to be widely employed in this sector, but other econometric methods have been used in past research. Therefore, the research article should thoroughly evaluate the relevant literature to identify the literature gap, highlight the need for further research to fill the gap, and discuss the potential contributions of the CS-ARDL approach to the area. As a whole, the study would contribute to what is already known about the factors that affect life expectancy, especially in Eastern Europe, and it would give policymakers and healthcare practitioners new information they can use to boost health in the region.

## 3 Methodology

### 3.1 Data

To determine the econometric result, secondary data was used. The World Bank's WDI and IMF were supplementary sources for this research. This analysis of life span aimed to show the power of public health policies. Economic and financial development growth can be measured by looking at financial development. Renewable energy, fossil fuels, education, CO_2_ emissions, and health expenditures are just some of the other control variables considered in this study to determine what and how much ultimately determines health outcomes. For a deeper dive into the data for 18 Eastern European countries from 1990 to 2021, see Table 1. Health outcomes in Eastern Europe are proxied by the dependent variable of life expectancy at birth, measured in years. All of the variables were logged in this study. Nonlinearity, heteroscedasticity, outlier, and skewness in the data can be dealt with using log transformations for variables in this study [37]. Improve model fit, lessen the impact of outliers, and make coefficients easier to comprehend with a log transformation [38].

**Table 1:** List of all variables.

Variables Name	Symbol	Unit	Source
Health Outcomes	lnHO	Total expected life span at birth (years)	World Development Indicator [39]
Health Expenditure	lnHEX	Current health expenditure per capita (current US$)	
CO_2_ Emission	lnCO_2_	CO2 emissions (metric tons per capita)	
Renewable energy	lnREN	Renewable energy consumption (% of total final energy consumption)	
Fossil Fuel	lnFOS	Fossil Fuel (% of total final energy consumption)	
Institutional quality index	lnIQI	Index constructed through PCA from governance indicators	WGI [40]
Financial Development	lnFD	Financial development index	IMF [41]

**Fig. 1:**
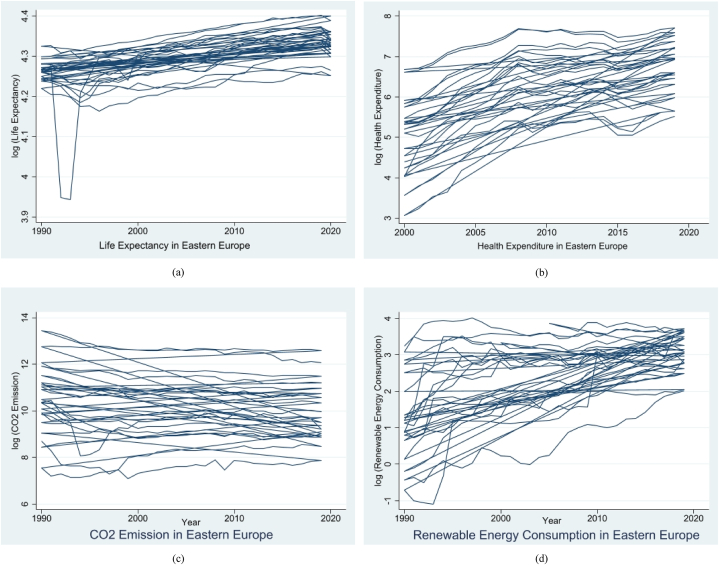
(a)Life expectancy in eastern Europe Fig. 1(b). Health expenditure in eastern Europe Fig. 1(c). Health expenditure in eastern Europe Fig. 1(d). Renewable energy consumption in eastern Europe.

Moreover, the graphical representation depicted in Fig. 1(a) and (b), 1(c), and 1(d) illustrates the yearly patterns of the examined parameters within the context of Eastern Europe.

Only CO2 emissions appear stable among the abovementioned metrics, while others seem to increase.

Table 2 presents the descriptive statistics for the variables from 1996 to 2021. The most important descriptive statistics for the paper's variables are shown in this summary table. The range of values for the natural logarithm of life expectancy (lnLE) variable is from 3.942 to 4.401, with a mean of 4.280. There are 588, 600, 525, 643, 605, and 646 observations for the variables lnHEX (natural logarithm of health expenditure), lnCO2 (natural logarithm of carbon dioxide emissions), lnIQI (institutional quality index), lnFD (natural logarithm of financial development), and lnREN (natural logarithm of renewable energy consumption). The means and standard deviations for these variables are (in order) 6.178, 10.13, 0.526, 0.451, 2.465, and 4.365, and (respectively) 0.904, 1.359, 0.124, 0.091, 0.964, and 0.277. In essence, these statistics give readers a clear view of the distribution of the variables employed in the research, which can aid in their understanding of the data's properties and interpretation of the study's findings.

**Table 2:** Summary statistics.

variables	N	mean	sd	min	max
lnLE	1003	4.280	0.049	3.942	4.401
lnHEX	588	6.178	0.904	3.062	7.705
lnCO2	600	10.13	1.359	7.098	13.44
lnIQI	605	0.026	0.724	−2.202	5.292
lnFD	643	0.451	0.091	0.231	0.556
lnREN	605	2.465	0.964	−1.094	4.025
lnFOS	646	4.365	0.277	2.569	4.604

### 3.2 Theoretical framework and model specification

Smith [20] suggests the following outline for the health production function:

(1) HO=f(MN)

Smith [20] demonstrated the relationship between medical and non-medical input combinations and the resulting output using Eq (1) to hypothesize a health production function. Therefore, health production depends not just on the healthcare system and its resource intake but also on non-medical, socio-economic, financial, and physical elements. Arawomo et al. [35] applied Smith's [20] general form of the health production function to analyze the dynamic relationship between economic growth, energy use, and infant mortality in Sub-Saharan African (SSA) countries. Non-medical, social, economic, and lifestyle variables are represented by the letter "N," while "M" stands for medical resources. The theory states that as healthcare costs rise, the quality of patient treatment will also rise. As a result, increasing funding for the medical sector may lead to improved access to healthcare for the general public. The rule of diminishing returns applies, however, in a different context.

A population's health's societal, economic, and physical contexts are also relevant metrics [37]. The theoretical framework for this study is Or's [42] health production function. He classifies environmental variables, lifestyle choices, and socio-economic status as the three main types of non-medical influences. Or [42] argues that drinking alcohol, eating poorly, not exercising, and not practicing good personal cleanliness are all significant contributors to poor health.

Three additional variables can influence health outcomes. These are income, education, and employment. Health and income levels are positively correlated. Increases in disposable income directly affect the quality of life in areas such as nutrition, housing, and access to quality education. Several researchers, including Preston [43], Winegarden [44], and Saunders [45], have found that the distribution of wealth has a significant effect on health. Health is another area in which education may improve lives. This is characterized as follows. Education influences the selection of a career, the choice of a healthy diet and the avoidance of unhealthy habits, the efficient utilization of medical care, etc. Here, the occupation has a substantial effect on health [46]. As total final consumption expenditure includes all goods, medical, and education expenditures, we use it as a proxy variable.

So, according to Or [42], the specific model for the study is.

(2) $${HO}_{t}=\alpha_{0}+\alpha_{1}M_{t}+\alpha_{2}N_{t}+{€}_{t}$$

Simplifying Eq. (2), we may refer to the health outcome (HO; measured by life expectancy), the medical variable (M; measured by healthcare expenditure), and the non-medical variables (N; measured by energy consumption, financial development, and pollution) as vectors, respectively. To time, the subscript t is time. Here, $\alpha_{0}$ represents the intercept coefficient, while $\alpha_{1}$ and $\alpha_{2}$ represent slope coefficients. The $\alpha_{0}$ remains constant throughout the period.

We can now establish connections between health outcomes and relevant medical and non-medical factors. Eq (3) provides a more refined version of the health outcome equation presented before.

(3) $${HO}_{t}=\beta_{0}+\beta_{1}HEX+\beta_{2}CO_{2}+\beta_{3}HDI{+\beta }_{4}FD+\beta_{5}FOS+\beta_{6}REN+{€}_{t}$$

Here, HOt stands for health outcome, HEX for health expenditure in general, IQI for the institutional quality index, and FD for financial development. Emissions of carbon dioxide (CO2), use of renewable energy sources (REN), and use of fossil fuels (FOS) are all indicated.

In Eq (4), all variables used in this analysis are shown in logarithmic form. Therefore, the estimated model is as follows:

(4) $${lnHO}_{t}=\beta_{0}+\beta_{1}lnHEX+\beta_{2}lnCO_{2}+\beta_{3}lnHDI{+\beta }_{4}lnFD+\beta_{5}lnFOS+\beta_{6}lnREN+{€}_{t}$$

Smith's and Or's models require medical and non-medical resources (M and N, respectively) as inputs. The variables in this paper are identical to those in their models, but we alter them using logarithms. This is a standard procedure in empirical studies since it increases confidence in the estimates. In addition to the traditional covariates, this paper's model additionally features a time trend variable that may be used to account for unexpected shifts in health outcomes. The medical and non-medical variables were generalized in Smith and Or's model. This paper's model detailed all of the potential variables. This research utilized health expenditure as the medical variable and a group of variables as the non-medical variable. The non-medical variables include pollution, IQI, financial development, and consumption of renewable and fossil fuels. This paper's model utilized diverse variables in non-medical variables, including environmental, economic, energy, and development-related variables.

### 3.3 Estimation technique and econometrics procedure

The data used in this analysis comes from the World Bank, WGI, and IMF. Health outcomes (HO) in 18 Eastern European countries are analyzed from 1990 to 2021 concerning energy, financial development, education, health expenditure, and pollution-related variables.

The panel data and cross-sectional association suggest that these Eastern European countries may be experiencing a stationary CSD, SH, or mixed-order stationary issue. The paper uses the CSD test because of the extensive cooperation and coordination among Eastern European nations. While most Eastern European economies are expanding, the growth rates differ considerably across the region. That's why we employ a measure for slope homogeneity. The second-generation unit root and cointegration tests must be used to confirm the CSD and SH problems. We utilize the CIPS and CADF (Pesaran [47]) CSD and SH monitoring assays. The article presents the cointegration test after the unit root test. In this study, a second-generation cointegration test was used [48]. The Westerlund [48] test considers the CSD, heterogeneous effects, and nonstationary data issues. After these safeguards were considered, the CS-ARDL method was implemented. Quantile regression estimations were also used in the study to evaluate the robustness. Fig. 1 depicts our entire research strategy. In Fig. 1, the left side shows the research's required methods and the right side shows the test's name. The left side shows the study applied CSD test, and the right side shows which specific tests the research applied.

**Fig. 2:**
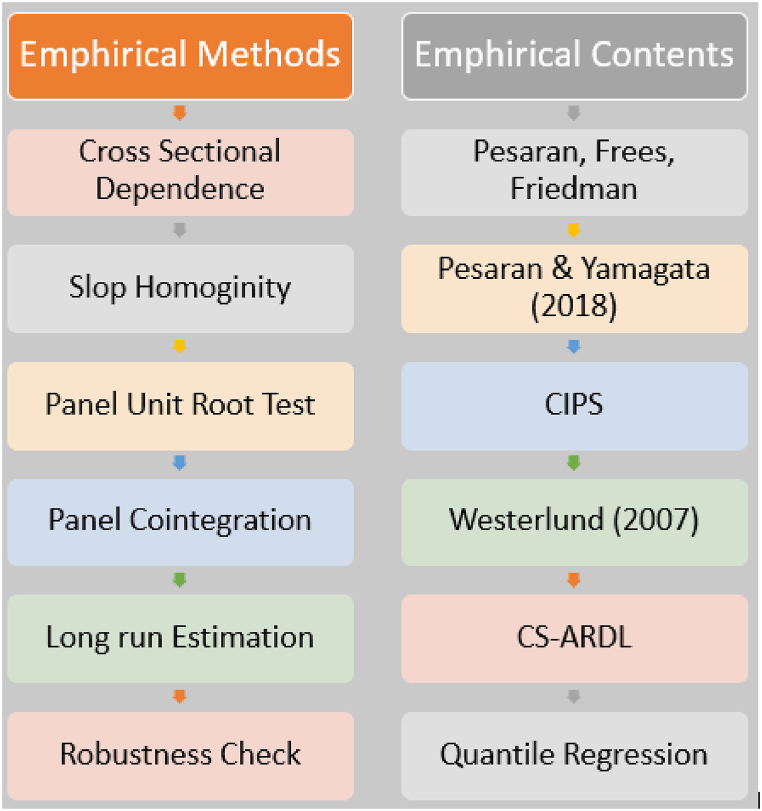
Steps of empirical methods.

The econometric techniques employed in this research are listed below in the Fig. 2.

#### 3.3.1 Cross-sectional dependence (CD) test

Due to the use of panel data in this investigation, it is essential to check the data for any indicators of CSD. Conditions of the exact socio-economic nature can also induce CSD. The execution of succeeding tests is dependent on the results of this test. As a result, the CSD test developed by Pesaran is utilized in this research [41]. The CSD equation can be represented by the Eqs that are shown below. (5).

(5) $$CSD=\sqrt{\frac{2T}{N\left(N-1\right)}}\left(\sum_{j=1}^{m-1}\;\sum_{i=j+1}^{m}{Ɵ}_{ji}^{t}\right)$$

Where N represents time and T represents a cross section. ${Ɵ}_{ji}^{t}$ Represents the estimation of the correlation residual.

#### 3.3.2 Slope homogeneity test

Equally significant is the conduct of SH. This test examines the data for commonalities. As a result, the Pesaran and Yamagata [41] test is applied. The SH equation is shown below in Eqs. (6) and (7).

(6) $$\Delta_{SH}^{∼}=N^{\frac{1}{2}}2k^{\frac{1}{2}}\left(\frac{1}{N}S^{∼}-k\right)$$

(7) $$\Delta_{SH}^{∼}=N^{\frac{1}{2}}\left(\frac{{2k{\left(T-k-1\right)}^{-}}^{\frac{1}{2}}}{T+1}\right)\left(\frac{1}{N}S^{∼}-k\right)$$

#### 3.3.3 Stationarity test

To determine the unit root of the data, CSD and SH perform an analysis of additional guidance. After confirming CSD and SH problem, the first-generation unit root tests will not be effective. The Cross-Section IPS (CIPS) analysis was used for this investigation. The degree of stationarity (level or first difference) can be determined by taking this exam.

#### 3.3.4 Cointegration test

After completing the unit root test, the panel cointegration test developed by Westerlund and Edgerton [49] is applied to evaluate the cointegration. The findings of this test are reliable, and it considers both CSD and SH when analyzing panel data. Therefore, the following Eqs (10), (11) are included in the equation that is used to conduct the cointegration test:

(8) $$G_{t}=\frac{1}{N}\sum_{j=1}^{N}\frac{{Ɵ}_{j}^{ƭ}}{SE{Ɵ}_{j}^{ƭ}}$$

(9) $$G_{a}=\frac{1}{N}\sum_{j=1}^{N}\frac{T_{j}^{ƭ}}{{Ɵ}_{j}^{ƭ}\left(1\right)}$$

(10) $$P_{t}=\frac{{Ɵ}_{j}^{ƭ}}{SE\left({Ɵ}^{ƭ}\right)}$$

(11) $${Ɵ}^{ƭ}=\frac{P_{a}}{T}$$

Here, Q^f^ shows the ratio of correction yearly.

#### 3.3.5 CS-ARDL methodology

Short-term and long-term relationships among energy use, CO_2_ emission, financial development, and health outcomes are calculated using the CS-ARDL model. The CS-ARDL is the final answer because it overcomes CSD and heterogeneity problems and is immune to endogeneity and non-stationarity [50]. The CS-ARDL strategy applies to this inquiry due to CSD and slope homogeneity problems. The CS-ARDL model can be seen in its broad outline in Eq. (12):

(12) $${\Delta lnLE}_{it}={Ɵ}_{i}+\sum_{j=1}^{m}{Ɵ}_{it}{\Delta lnLE}_{i,t-j}+\sum_{j=0}^{m}{Ɵ}_{ij}^{\prime }+AEV_{i,t-j}+\sum_{j=0}^{m}{Ɵ}_{it}^{\prime }{\bar{Z}}_{t-j}+{€}_{i,t}$$

It denotes the cross-section average and which is equivalent to ($\left(\Delta {\bar{lnLE}}_{t},{{\bar{AEV}}_{t}}^{\prime }\right)$.' Where AEV represents all independent variables in this study.

#### 3.3.6 Robustness check (quantile regression)

Applying conventional methods may provide preliminary estimates for cross-sectional dependency pitch heterogeneity. However, when the relationship between the two variables is not linear, or when the data is skewed or includes outliers, quantile regression can still be used to determine the strength of the relationship between the two variables. Unlike ordinary least squares regression, Quantile regression calculates the dependent variable's conditional median or any other quantile given the independent variables.

The mean is vulnerable to outliers, so having an alternative measure of significant trends, such as the median or other quantiles, is helpful. For instance, a dataset's mean income may be inaccurate because it may be skewed by a few highly well-off individuals, making it a poor proxy for the "typical" income of the population as a whole. Quantile regression can then be used to obtain more accurate approximations of the population median or other quantiles.

## 4 Results

The results of CSD are presented in Table 3, which also illustrates the interconnectedness of the variables. In other words, according to the results of the Pesaran CSD test, the Frees test, the Friedman test, and the Pesaran abs test, the null hypothesis that assumes no cross-sectional interdependence is rejected. According to the findings of the CSD test, the null hypothesis ought to have been disregarded at the 1 % significance level. This substantiates the claim that the dataset includes cross-sectional dependence. Similar social and economic strategies explain this cross-sectional dependence.

**Table 3:** Results of CSD test.

	H_0_: There exists a cross-sectional dependence	
	Test statistics	P-value
Pesaran CSD	4.259	0.012
Frees test	2.015	Alpha = 0.01: 0.234
Friedman test	124.025^a^	0.001
Pesaran abs	42.245**	0.004

a And * shows 1 % and 10 % significance, respectively.

The results of the homogeneity test for slopes are shown in Table 4. For the sake of this analysis, let's pretend that the slope values are constant. The findings show that the delta title is statistically significant at the 5 % level, while the delta title adjusted is significant at the 1 % level. Therefore, the model considers heterogeneity and rejects the null hypothesis that slope value distributions are uniform.

**Table 4:** Slope homogeneity test.

Slope homogeneity tests Δ statistic P-value		
Δˇ test	7.256***	0.012
${\Delta ˇ}_{adj}$ test	9.254***	0.000

The results of the CIPS unit root test are summarized in Table 5. As the empirical CIPS unit test result shows, lnIQI, lnCO2, lnREN, and lnFD all have unit root issues. Once the first difference is taken, the variables are integrated into I (1) and become significant at the 1 % level. At the 1 % significance level, the data show that the levels and first differences of lnCO2, lnIQI, lnHEX, and lnREN are all different. Therefore, it has been decided that lnCO_2_, lnIQI, lnHEX, and lnREN will be incorporated into I (0).

**Table 5:** Second-generation unit root test.

Variables	Level		First difference		Order
	Without trend	With trend	Without trend	With trend	
Cross-Sectionally Augmented IPS (CIPS)					
lnLE	−0.970	−1.297	−3.639***	−3.636***	I (1)
lnCO_2_	−2.727***	−2.809*	−3.715***	−3.706***	I (0)
lnIQI	−2.021**	−2.256***	−3.542***	−5.023***	I (0)
lnFD	−1.633	−1.791	−2.066***	−2.293***	I (1)
lnHEX	−2.895***	−4.023***	−5.730***	−5.124***	I (0)
lnREN	−2.886***	−3.024*	−4.839***	−5.038 ***	I (0)
lnFOS	−1.381	−1.956	−3.257***	−4.485***	I (1)

Values in parentheses indicate P-values, and the superscripts *, **, and *** denote a 10 %, 5 %, and 1 % degree of significance, respectively.

The results of the error correction test given by Westerland [47] are analyzed using Bootstrap p-values to ascertain whether or not the series used in the analysis are dependent across periods [51]. Group tau (Gt), Group alpha (Ga), Panel tau (Pt), and Panel alpha (Pa) crucial values are used in the interpretation of the results to account for the heterogeneity between the series [52]. Table 6 displays the results of the cointegration test. The null hypothesis is the absence of long-run cointegration between the dependent variable and the independent factors. The null hypothesis is denied at a 1 % significance level, as shown in Table 6, because Gt and Pt have extremely small p-values. Therefore, this analysis indicates that health results in Eastern European are long-term cointegrated with other independent variables.

**Table 6:** Results of Westerlund test for cointegration.

Variables	Value	Z- value	P-value
Gt	−3.125	4.523	0.001
Ga	−0.746	1.275	0.585
Pt	−4.256	4.259	0.005
Pa	−0.998	4.256	0.856

Findings from the CS-ARDL are shown in Table 7. A robust positive association exists between health spending and health outcomes (life expectancy). A 1 % rise in health expenditure will improve health outcomes by 0.0314 % in the long run and reduce by 0.0271 % in the short run, according to the coefficients of lnHEX. The long-run effect is significant, but the short-run outcome is insignificant. The findings from both the quantile regression corroborate the findings. The results indicate that life expectancy will benefit from the government's decision to raise general health expenditure and the number of health-related services such as hospitals, clinics, and health care services. The findings are similar to Shahraki [53] in Iran and Polcyn et al. [54] in Asia. Results from the CS-ARDL and QR all point to a negative association between CO_2_ emissions and life expectancy in the short and long run. The long-term lnCO_2_ coefficient is −0.026, and the short-term lnCO_2_ coefficient is −0.0391; only the long-run coefficient is statistically significant at the 1 % level. So, a 1 % rise in CO_2_ is associated with a 0.026 % decrease in long-term life expectancy. The findings are similar to Voumik et al. [55], Ghosh et al. [56], Sultana et al. [57], and Azam et al. [58]. CO_2_ emissions significantly contribute to atmospheric pollution, adversely affecting human health. Air pollution from CO2 emissions is linked to respiratory illnesses and a shortened lifespan. Heat-related illnesses and fatalities are becoming more common due to rising global temperatures caused by CO2 emissions [59]. There is a long- or short-term correlation between IQI and health outcomes. A 1 % rise in IQI will improve health outcomes by 0.0814 % in the long run and 0.0411 % in the short run, according to the coefficients of lnIQI. Popescu [60] and Nuhu et al. [61] got the same outcome. A clear correlation between economic development and health outcomes was found, but this effect was insignificant. In particular, the lnFD coefficient is positive at 0.0551 and 0.0145 in the long and short run, but the impact is insignificant. According to the CS-ARDL results, a statistically significant favorable association exists between renewable energy consumption and life expectancy in the long run. Long-term and short-term coefficients of lnREN are 0.0214 and −0.0219, respectively. Indicate that a 1 % increase in renewable energy consumption is associated with a 00.0214 % improvement in health outcomes, respectively, at the 1 % significance level. Results are consistent with those of Majeed et al. [62] and Voumik et al. [63]. On the other hand, there is a statistically significant negative association between fossil fuel consumption and health outcomes. Long-term and short-term coefficients of lnFOS are −0.135 and 0.026, respectively, indicating that a 1 % increase in fossil fuel consumption is associated with a 0.135 % and a 0.026 % reduction in health outcomes, respectively, at the 1 % level of significance in the long run. The result was the same for Ibrahim [64] and Lelieveld et al. [65].

**Table 7:** CS-ARDL.

	CS-ARDL	
VARIABLES	Long-Run	Short-run
L.lnLE		−0.314 (0.287)
lnHEX	0.0314*(0.0162)	−0.0271 (0.0135)
lnCO_2_	−0.026**(0.0126)	−0.0391*(0.0218)
lnIQI	0.0814***(0.00923)	0.0411**(0.0109)
lnFD	0.0551 (0.0574)	0.0145 (0.0882)
lnREN	0.0214***(0.0053)	−0.0219*(0.0123)
lnFOS	−0.1346*(0.0775)	−0.0263 (0.111)
Observations		264
Number of groups		15
R-squared		0.139

Standard errors in parentheses.

***p < 0.01, **p < 0.05, *p < 0.1.

Table 8 demonstrates the robust results of this paper utilizing the quantile regression. This quantile regression result shows the impact of health expenditure, environmental pollution, education, renewable energy, fossil fuels, and GDP on life expectancy at low to high quantiles (0.05–0.95). The health expenditure positively influences life expectancy at low to high quantiles (0.05–0.75), and the coefficients are 0.129, 0.0726, 0.129, and 0.0286. Still, the high quantile shows a negative relationship between health expenditure and life expectancy. CO_2_ emission also negatively impacts life expectancy at low to the highest quantiles (0.05–0.95), and the coefficients are −0.226, −0.253, −0.226, −0.251, and −0.201, respectively. The IQI positively impacts life expectancy at all quantiles but is only significant in Q25 and Q50. The GDP suggests a positive effect at all quantiles on life expectancy, but the lower quantiles' coefficients are insignificant. Only the top two quantiles are significant.

**Table 8:** Quantile regression.

VARIABLES	Q5	Q25	Q50	Q75	Q95
lnHEX	0.129**	0.0726	0.129**	0.0286	−0.120***
	(0.0546)	(0.0641)	(0.0549)	(0.0421)	(0.0412)
lnCO_2_	−0.221***	−0.251***	−0.226***	−0.251***	−0.201***
	(0.0355)	(0.0330)	(0.0355)	(0.0245)	(0.0258)
lnIQI	0.0228	0.104***	0.0148**	0.0121	0.0124
	(0.0321)	(0.0202)	(0.0217)	(0.0150)	(0.0158)
lnFD	0.00608	0.0180	0.00608	0.0632*	0.149***
	(0.0513)	(0.0147)	(0.0513)	(0.0354)	(0.0373)
lnREN	0.364***	0.260***	0.364***	0.383***	0.276***
	(0.0490)	(0.0455)	(0.0490)	(0.0338)	(0.0356)
lnFOS	−0.426***	−0.257***	0.259***	0.153***	−1.190***
	(0.139)	(0.129)	(0.139)	(0.0957)	(0.246)
Constant	1.690***	0.0875	1.562***	1.542***	1.254***
	(0.379)	(0.452)	(0.457)	(0.330)	(0.324)
Observations	452	452	452	452	452

Standard errors in parentheses.

***p < 0.01, **p < 0.05, *p < 0.1.

Renewable energy positively impacts life expectancy at all quantiles; all coefficients are significant. Fossil fuel consumption negatively impacts life expectancy at lower and higher quantiles. On the other hand, medium quantiles show a positive impact. All coefficients are significant.

## 5 Discussion

Findings show that when health expenditure increases, life expectancy rises. Similar results can be found in Ref. [66]. In Eastern European countries, health expenditure and structural development improved recently, which pushes people'speople's health outcomes [67]. Spending on healthcare can significantly affect life expectancy by increasing access to medical care, decreasing the prevalence of illness, and fostering the adoption of preventative practices. Because of more healthcare, Eastern Europeans now get the checkups, screenings, and therapy needed for acute and chronic illnesses. With more money going toward healthcare, patients will have greater access to cutting-edge diagnostic tools, therapeutics, and surgical procedures. Spending on healthcare has the added benefit of facilitating illness prevention and early diagnosis. Spending on healthcare has the added benefit of funding new medical studies and innovations. This has the potential to pave the way for the creation of innovative pharmaceuticals, therapeutics, and medical procedures, all of which can boost health results and, by extension, life expectancy. Conversely, CO2 emissions adversely affect both short- and long-term health in Eastern Europe. Recently eastern European countries' governments have focused on the environment, and that help to improve public health [68,69]. However, Eastern European countries' institutional quality positively impacts health. Institutional quality is the extent to which a society's institutions provide access to and deliver essential public services, including healthcare, education, and infrastructure. Institutions that meet these three criteria are considered the highest quality. They can also enforce market regulation and safeguard citizens' rights [70,71]. There is accumulating data indicating a link between better health outcomes, longer life expectancies, and higher standards of care institutions provide. Several hypotheses suggest a positive relationship between health outcomes, life expectancy, and high-quality institutional support. First, people are more likely to receive excellent care from reputable hospitals. This is so because these jurisdictions can better recruit and retain qualified medical personnel, invest in cutting-edge healthcare technology, and police the industry to benefit patients. Second, public health programs are more likely to receive funding from prestigious institutions. Diseases can be avoided, healthier lifestyles encouraged, and easier access to medical treatment made possible by such initiatives. Third, the rights of citizens are more likely to be safeguarded by strong institutions. The availability of clean water and food and the opportunity to further one's education fall under this category. These things can majorly affect how well you feel and how long you live [[72], [73], [74]]. This research also indicated that expanding financial development in Eastern Europe benefited health outcomes. The findings are consistent with [75,76] results. A large government budget and tax money come hand in hand with high financial development. Eastern European governments invested a large percentage of the funds in public health, infrastructure, and research areas. Countries with high per capita incomes and excellent administration and governance may have greater food security. This ensures that the populace will not fall victim to food poisoning or dysentery because of the food and water readily accessible to them. Increased spending on public sanitation contributes to eradicating malaria and other illnesses [77,78]. The research also shows that increasing the overall usage of renewable energy is good for public health in Eastern Europe. The results were consistent with those reported in Refs. [[79], [80], [81]]. Eastern European countries are switching to renewable energy, and they got health benefits [82]. Thus, sustainable energy consumption has surpassed non-renewable energy consumption in Eastern Europe, benefiting human health. Renewable energy improves environmental quality by progressively replacing traditional energy sources, increasing life expectancy, decreasing infant mortality, and preventing occurrences of tuberculosis. The findings also suggest that burning fossil fuels risks human health in Eastern Europe [83,84]. Similar results were found in these studies [[85], [86], [87]]. Pollutants like PM2.5, NOx, and SO2 are released into the air when fossil fuels are burned. Lung cancer and heart disease are two breathing illnesses that can result from prolonged exposure to these toxins. Using fossil fuels is a significant cause of global warming, which has many potentially harmful effects on human health. Air pollution, climate change, water pollution, and workplace dangers are just some of how burning fossil fuels can negatively impact human health. These influences may skew or even reduce life span [[88], [89], [90]].

## 6 Conclusion

This study examines the connections between the environment, energy, and health in Eastern Europe. Among the factors that significantly affect longevity in Eastern Europe are economic growth, health care spending, carbon dioxide emissions, strong institutions, renewable power sources, and traditional energy sources. The analysis uses econometric tools of the second generation. The cointegration technique developed by Westerland was used to assess the long-run equilibrium connection between the variables, and the slope homogeneity test developed by Pesaran and Yamagata was used to determine the homogeneity of slope values. Estimates from Pesaran and Smith and Chudik and Pesaran are used in this study to illustrate the CS-ARDL technique. Westerland states that the ARDL technique must be used if the panel dimensions are significantly larger than the transversal components (T > N), which is the situation here.

The research also confirmed the existence of CSD and the heterogeneity of the data. In addition, the CIPS Pesaran (2007) unit root analysis found a mixed integration order of the investigated variables, which is relevant to the second iteration of cointegration methods. Except for population, all other variables were found to be cointegrated with health results over the long term. Estimates from the quantile regression techniques are consistent with those from the CS-ARDL. This research showed that health expenditure, institutional quality, and renewable energy consumption in certain Eastern European countries improved health outcomes between 1997 and 2022. One of the primary reasons for discovering a clear correlation between renewable energy consumption and health outcomes is the increased use of green and environmentally friendly resources over non-renewable resources. The results also show that CO_2_ emissions have a detrimental effect on health. Similarly, fossil fuel consumption can distort health outcomes in Eastern Europe. At last, this research established a causal link between healthcare costs and beneficial effects. Spending more money on public health facilities is one way to improve overall health. The study also suggests essential policies based on the findings.

## 7 Policy recommendation

➢ Eastern European policymakers should prioritize raising healthcare budgets, enhancing the effectiveness of healthcare delivery, and boosting people's access to healthcare. These changes will increase the standard of care available in the area, increasing life expectancy [91]. The Polish government, for instance, has increased funding for the National Health Fund and introduced new tax advantages for healthcare professionals, among other reforms, to raise healthcare spending. The Romanian government has introduced new payment structures for healthcare providers and streamlined the procurement process as part of a series of measures intended to increase efficiency in healthcare delivery. The Bulgarian government has increased the availability of primary care physicians and built new hospitals in remote places to serve its citizens better [92]. Other Eastern European countries should increase their health budgets and revise health policies. These are only a handful of the many policy suggestions that can be implemented to boost Eastern European healthcare budgets.
➢ Pollution and CO2 emission reduction should be top priorities for Eastern European policymakers. These changes will increase life expectancy by improving air quality and decreasing the prevalence of chronic diseases [93,94]. The government of Slovakia, for instance, has enacted several policies, such as a carbon tax and investments in renewable energy, to reduce the country's CO2 emissions. Improvements in building insulation regulations and the introduction of energy-efficient equipment are just two examples of the measures adopted by the Romanian government to increase energy efficiency. New solar and wind farms are among the many renewable energy investments the Hungarian government has made in a series of reforms [95,96]. These are only a handful of the many possible policy prescriptions for lowering CO2 emissions in Eastern Europe.
➢ Eastern European policymakers should work to improve openness and accountability, increase spending on public services like education and healthcare, and foster a culture of institutional quality. These changes will boost life expectancy by increasing the reliability of regional institutions [[97], [98], [99]]. The Polish government, for instance, has introduced new anti-corruption legislation and appointed new judges to the Supreme Court to reinforce the rule of law. The Romanian government has enacted several measures to increase openness and accountability, such as boosting public engagement in decision-making and expanding access to government information. The Bulgarian government has increased the number of teachers and nurses, expanded access to healthcare in rural areas, and enacted other measures to invest in these sectors. These are only a handful of the many possible policy prescriptions for enhancing Eastern Europe's institutional quality.
➢ Cleaner air and longer lifespans are two benefits of the widespread adoption of renewable energy sources like wind, solar, and hydroelectric. Governments can set renewable energy goals to encourage the use of renewable energy and reduce reliance on fossil fuels. Policies like feed-in tariffs and renewable portfolio standards can be implemented to promote investment in renewable energy and reach targets for the share of electricity generated from renewable sources by a specific year. Governments can encourage the construction of wind farms, solar farms, and hydropower facilities to increase the availability of sustainable energy. Subsidies and tax credits, an accelerated permitting process, and low-interest loans are all measures that could be taken to encourage the growth of green energy. Improving air quality and reducing the health risks associated with climate change will be significantly aided by a greater reliance on green energy sources [[100], [101], [102]]. Increased use of clean energy sources has been linked to longer life expectancy, so governments should prioritize setting renewable energy goals, promoting renewable energy infrastructure, encouraging energy-saving measures, and educating the public about renewable energy.
➢ The health impacts of climate change on humans can be mitigated, and air quality improved by reducing the use of fossil fuels. To lessen the burden on the environment, governments should promote eco-friendly modes of transit like electric vehicles, public transportation, cycling, and walking. Policies like congestion fees, fuel taxes, and electric car subsidies could help accomplish this goal. In addition, governments can aid in reducing air pollution and improving respiratory health, which in turn can increase life expectancy by encouraging sustainable modes of transit [[103], [104], [105]]. Finally, Eastern European governments are interested in lowering fossil fuel usage and raising life expectancy. In that case, they must prioritize the spread of clean energy, encourage sustainable transportation, implement energy efficiency measures, and fund public transportation infrastructure.

## 8 Limitations and future research

The CS-ARDL method relies on a lengthy time-series collection, which may be unavailable in some regions. Results could be skewed if data were present or complete. There may be a bidirectional causal connection between the study's variables if they are endogenous. For example, a high life span may attract more foreign direct investment (FDI) and vice versa. Life expectancy is affected by many variables, some of which were not taken into consideration in this research. These include access to healthcare, nutrition, and lifestyle choices. Therefore, omitted variable bias may exist in the calculated coefficients. Because of its linear assumptions, the CS-ARDL method may fail to convey the nonlinearities and complexities of real-world relationships. Healthcare spending, environmental pollution, and social factors are all potential additional elements to consider in future life expectancy studies. Future studies may employ non-linear models in light of the non-linear and complex connection between the variables. In the future, researchers may want to compare life expectancy statistics from various countries to learn more about the factors that make a difference. To determine what factors affect one's life expectancy, researchers in the future may use causal analysis methods like instrumental variables and natural trials.

## Funding statement

Any entity did not fund the research.

## Consent for publication

N/A.

## Data availability statement

The data is available in this link: Nica, Elvira (2023), “Health Expenditure, CO2 Emissions, Institutional Quality, and Energy Mix on Life Expectancy in Eastern Europe”, Mendeley Data, V1, https://doi.org/10.17632/k6v29rvdh5.1.

## CRediT authorship contribution statement

**Elvira Nica:** Conceptualization, Formal analysis, Funding acquisition, Investigation. **Adela Poliakova:** Methodology, Project administration, Resources, Validation. **Gheorghe H. Popescu:** Funding acquisition, Resources, Supervision, Visualization, Writing – original draft. **Katarina Valaskova:** Project administration, Resources, Software, Writing – review & editing. **Stefan Gabriel Burcea:** Data curation, Project administration, Software, Validation, Writing – original draft. **Andreea-Ligia Drugau Constantin:** Data curation, Investigation, Software, Visualization, Writing – review & editing.

## Declaration of competing interest

The authors declare that they have no known competing financial interests or personal relationships that could have appeared to influence the work reported in this paper.

## Appendix A Abbreviations

AMG: Augmented Mean Group
CIPS: Cross-Sectional IPS
CO_2_: Carbon dioxide
CS-ARDL: Cross-sectional Autoregressive Distributed Lag
CSD: Cross-Sectional Dependence
EG: Economic Growth
EU: European Union
FD: Financial Development
GDP: Gross Domestic Product
GHG: Greenhouse Gas
HO: Health Outcomes
NO_2_: Nitrogen Dioxide
OECD: Organization for Economic Cooperation and Development
OLS: Ordinary Least Squares
PPP: Purchasing Power Parity
POLL: potential years of life lost
QR: Quantile Regression
REC: Renewable Energy Consumption
SH: Slope Homogeneity
WHO: World Health Organization

## Appendix B List of Eastern European Countries-

Albania, Belarus, Bosnia and Herzegovina, Lithuania, Moldova, Poland, Romania, Serbia, Slovakia, Slovenia, Ukraine, Bulgaria, Croatia, Cyprus, the Czech Republic, Estonia, Hungary, and Latvia.
